# Expression and Subcellular Targeting of Human Complement Factor C5a in *Nicotiana species*

**DOI:** 10.1371/journal.pone.0053023

**Published:** 2012-12-28

**Authors:** Henrik Nausch, Heike Mischofsky, Roswitha Koslowski, Udo Meyer, Inge Broer, Jana Huckauf

**Affiliations:** 1 Department of Agrobiotechnology, Agricultural and Environmental Faculty, University of Rostock, Rostock, Germany; 2 Bioserv GmbH, Rostock, Germany; Cinvestav, Mexico

## Abstract

We evaluated transgenic tobacco plants as an alternative to *Escherichia coli* for the production of recombinant human complement factor 5a (C5a). C5a has not been expressed in plants before and is highly unstable *in vivo* in its native form, so it was necessary to establish the most suitable subcellular targeting strategy. We used the strong and constitutive CaMV 35S promoter to drive transgene expression and compared three different subcellular compartments. The yields of C5a in the T_0_ transgenic plants were low in terms of the proportion of total soluble protein (TSP) when targeted to the apoplast (0.0002% TSP) or endoplasmic reticulum (0.0003% TSP) but was one order of magnitude higher when targeted to the vacuole (0.001% TSP). The yields could be increased by conventional breeding (up to 0.014% TSP in the T_2_ generation). C5a accumulated to the same level in seeds and leaves when targeted to the apoplast but was up to 1.7-fold more abundant in the seeds when targeted to the ER or vacuole, although this difference was less striking in the better-performing lines. When yields were calculated as an amount per gram fresh weight of transgenic plant tissue, the vacuole targeting strategy was clearly more efficient in seeds, reaching 35.8 µg C5a per gram of fresh seed weight compared to 10.62 µg C5a per gram fresh weight of leaves. Transient expression of C5aER and C5aVac in *N. benthamiana*, using MagnICON vectors, reached up to 0.2% and 0.7% of TSP, respectively, but was accompanied by cytotoxic effects and induced leaf senescence. Western blot of the plant extracts revealed a band matching the corresponding glycosylated native protein and the bioassay demonstrated that recombinant C5a was biologically active.

## Introduction

Autoimmune and inflammatory diseases (AIIDs) are characterized by an overactive immune system. They are becoming more prevalent in society and more of a significant challenge to health authorities around the world. Particularly sepsis following bacterial infection often fail standard medical treatments due to the spread of antibiotic resistance [1]. With mortality rates exceeding 70%, sepsis is one of the top 10 causes of death worldwide [1]–[4]. Therefore, there is a strong demand for novel therapeutic approaches to regulate the immune system. Complement factor 5a (C5a) is a promising candidate for immunomodulatory therapies of sepsis [4]–[7], since it has been characterised as potent mediator of the innate immune system to infection and ‘key’ mediator of sepsis and septic organ dysfunction [1], [4]–[12]. Though, the development of this strategy is hampered by the lack of an efficient production system for recombinant C5a.

Recombinant pharmaceutical proteins are usually produced by fermentation in *Escherichia coli*, yeast, insect cells or mammalian cells, each with particular advantages and disadvantages often depending on the importance of post-translational modifications for therapeutic efficacy [13]. C5a produced in *E. coli* accumulates as inclusion bodies and therefore requires laborious solubilisation and refolding to achieve the native confirmation [14]–[16]. Attempts to express soluble C5a in *E. coli* have only been partially successful [17]. The solubilisation of inclusion bodies is undesirable in commercial downstream processing because of the increased process time and costs [18], therefore *E. coli* is not suitable for the commercial production of C5a.

In contrast to microbes, plants can fold and modify complex human proteins and should therefore be able to produce C5a in a soluble and active form [19]. Plants also have the advantage of economy, scalability and increased safety compared to animal cells, since they do not support the replication of human pathogens [20], [21]. Among the many plant species used for the production of recombinant proteins, tobacco (*Nicotinana tabacum*) is regarded as a major emerging platform for the production of certain pharmaceutical products, especially subunit vaccines and antibodies [22]–[24]. Tobacco is a leafy crop that produces up to 100 tons of leaf biomass per hectare and the total soluble protein (TSP) content is higher than in many other plant species [19]. Tobacco is neither a food nor a feed crop, thus reducing the likelihood of transgenic material contaminating the food or feed chain. Tobacco has also proven to be compatible with the demands of good manufacturing practice (GMP), which is critical for the regulatory approval of plant-derived pharmaceuticals [25]. Proof of concept has been demonstrated for various antibodies, subunit vaccines, hormones and enzymes, including other blood factors which have successfully been produced in tobacco with a yield of up to 0.15% TSP in leaves [19]. Several plant-derived pharmaceutical proteins have successfully completed phase I clinical trials to demonstrate safety, and many plant derived vaccines have also demonstrated efficacy by inducing a significant immune response [23], [26].

Our objective was to demonstrate the use of tobacco to produce recombinant human C5a. We compared different targeting strategies for their impact on protein yields, since it has been demonstrated in many previous studies that targeting proteins to different tissues and subcellular compartments is more successful than letting them accumulate in the cytosol or testing a single tissue [27]. We therefore compared proteins targeted to the apoplast, endoplasmic reticulum (ER) and protein storage vacuoles (PSVs) in leaves and seeds. We selected the commercial tobacco cultivar *N. tabacum* cv. Geudertheimer as the production host because of its superior biomass yield. In addition, as alternative to stable transformation, we investigated the feasibility of the MagnICON-based transient expression system for the expression of C5a in *N. benthamiana*.

## Materials and Methods

### Construction of plant expression vectors

We designed a synthetic C5a coding region based on the 74-amino-acid sequence of the human complement factor C5a, a cleavage product of the precursor protein C5 (accession no. P01031|678aa/Fig. S2). This was codon optimized for expression in tobacco (http://www.kazusa.org). The coding region was supplemented with a 28-amino-acid N-terminal ER-targeting peptide from the human IL6 gene (accession no. P05231-1) as shown in figure S1. We also added three codons (GCT TCC TCC) after the ATG initiation codon to improve the efficiency of translation [28]. These constructs were synthesized by the DNA Cloning Service (Hamburg, Germany).

We used the binary transformation vector pLH9000 (accession no. AF458478 [29]) containing the neomycin phosphotransferase type II gene (*npt*II) for selection [30] and ColE1 and VS1 origins of replication for propagation in *E. coli* and *Agrobacterium tumefaciens*, respectively. We inserted a polylinker and expression cassette comprising the CaMV 35S promoter with double enhancer [31], the tobacco mosaic virus (TMV) Ω-fragment [32] and the CaMV terminator [31] at the SfiI site, and then integrated the abovementioned synthetic gene constructs at the BamHI/EcoRI sites in the polylinker. The protein expressed using this basic cassette was targeted to the apoplast. For ER and vacuolar targeting, synthetic oligonucleotide sequences were designed based on the SEKDEL ER-retention signal [33], and the vacuole sorting determinant AFVY from phaseolin [34] as shown in figure S3. The synthesized oligonucleotides (Invitrogen) were fused to the 3′-end of the coding region at the MunI/BsrgI sites. All vectors were verified by DNA sequencing (GATC Biotech AG, Konstanz/Germany).

We used transient expression vectors based on *Tobacco mosaic virus* (cr-TMV/TVCV) provided by Prof. Dr. Yuri Gleba and Dr. Anatoli Giritch (Nomad Bioscience; Halle/Saale, Germany). These are derivatives of pICH18711, which has been optimized for high yields [35]. The pICH18711 vector is similar to pICH29912 except the green fluorescent protein (GFP) coding region has been inserted into the BsaI cloning site of pICH29912.

The coding region of C5aER and C5aVac was incorporated together with flanking BsaI restriction sites into the vector pLC by DNA Cloning Service Hamburg. The coding regions were inserted into the BsaI site of pICH29912 as described [36]. The vectors were verified by sequencing with primer pairs TMV-fw and TMV-rv (Table 1). The transient expression vectors were introduced into *Agrobacterium tumefaciens* strain ICF 320, a disarmed, auxotrophic derivative (ΔcysKa, ΔcysKb, ΔthiG) of strain C58 [37].

**Table 1:** Primers used for PCR and RT-PCR analysis.

Forward-Primer		Reverse Primer		Product length
Name	Sequence	Name	Sequence	
NtC5a-fw	5′-CCTGCTGCATTTCCTGCGAC-3′	NtC5a-rv	5′-ACGACACAGCACTCTGTGAAGG-3	166 bp
NtC5a-fw	5′-CCTGCTGCATTTCCTGCGAC-3′	NtC5aER-rv	5′-GCTCATCCTTCTCAGACAGTC-3′	268 bp
NtC5a-fw	5′-CCTGCTGCATTTCCTGCGAC-3′	NtC5aVac-rv	5′-GTACACGAAAGCCAGTCTG-3′	268 bp
2npt-fw	5′-TCCGGCCGCTTGGGTGGAGAG-3′	2npt-rv	5′-CTGGCGCGAGCCCCTGATGCT-3′	450 bp
Actin-fw	5′-GCAACTGGGATGATATGGAGAA-3′	Actin-rv	5′-GTGCCTTTGCAATCCACATCTG-3′	850 bp
TMV-fw	5′-GATCCGGACGTCGAAGGTTTCGAAGG-3′	TMV-rv	5′-CTTGACTCTAGCTAGAGCGGCCGCTGG-3′	977 bp/971 bp*

* pICH29912-C5aER and pICH29912-C5aVac.

### Stable transformation of tobacco plants

Wild type tobacco (*Nicotiana tabacum* cv. Geudertheimer) seeds were surface sterilized in saturated calcium hypochlorite solution and 0.1% Triton X-100 for 5 min. The seeds were rinsed several times with sterilized distilled water to remove the detergent and allowed to germinate on 4.4 g/l Linsmaier and Skoog (LS) medium including vitamins (catalog no. L0230.0050; Duchefa, Belgium) supplemented with 30 g/l sucrose and 6.5 g/l plant agar (catalog no. P1001.1000; Duchefa, Belgium) and adjusted to pH 5.7. The plants were maintained at 24/22°C day/night temperature with a 16-h photoperiod.

Tobacco leaves approximately one month old were used for *Agrobacterium*-mediated transformation essentially as described [38] but optimized for the transformation of cultivar Geudertheimer by Tina Hausmann (personal communication). Regenerated shoots were selected on LS medium containing 100 µg/ml kanamycin and 500 µg/ml cefotaxim. Regenerated plants were transferred to peat soil in the greenhouse until they were mature. Transgene integration was confirmed by PCR (Table 1).

### Transient expression in tobacco leaves

Transient expression in *N.* plants (6–9 weeks old) was carried out as described by Giritch *et al*. [39]. A bacterial smear was inoculated into 5 ml starter culture containing 50 µg/ml rifampicin and 50 µg/ml kanamycin, and was incubated overnight 28°C, 220 rpm. The overnight culture was sedimented (10 min, 4500 rpm, 4°C) and the pellet was resuspended in 50 ml infiltration buffer containing 10 mM MES (pH 5.5) and 10 mM MgCl_2_ (1∶100 dilution).

### DNA analysis

The T-DNA cassette was detected by PCR analysis of crude leaf extracts prepared from 100 mg of leaf tissue homogenized under liquid nitrogen and resuspended in 200 µl of extraction buffer (50 mM NaOH, 0.25% SDS). After boiling for 10 min and pelleting in a bench centrifuge, the supernatant was diluted 1∶5 in distilled water. PCR was used to detect both the C5a gene and the *npt*II marker. After an initial denaturation step (95°C for 5 min) we carried out 39 amplification cycles (95°C for 1 min, 58°C for 1 min, 72°c for 2 min) and a final elongation step (72°C for 10 min). The primer pairs are listed in Table 1.

### RNA analysis

Total RNA was isolated from 100 mg tobacco leaf tissue using Trizol reagent according to the manufacturer's instructions (Invitrogen). RNA integrity was assessed by visualizing the 28S and 18S rRNA bands under UV light in a denaturing 0.8% MOPS-agarose gel containing ethidium bromide. For reverse transcription (RT)-PCR analysis, total RNA samples were digested with DNase for 3 h and the removal of DNA confirmed by PCR against the endogenous *actin* sequence, which generates different products from genomic DNA and cDNA templates [40]. We used the RevertAid™ H Minus First Strand cDNA Synthesis Kit (Fermentas, St. Leon-Rot, Germany) according to the manufacturer's recommendations, with each reaction comprising 1 µg of DNase-treated RNA, 10 mM dNTP mix, 0.5 µg oligo(dT)-primer, the supplied 1× reaction buffer and 200 U reverse transcriptase. The reaction was incubated at 42°C for 60 min then stopped by heating to 70°C for 10 min. The PCR was carried out using the same parameters described for DNA amplification, but we used multiplex conditions including *actin*-specific primers so that transgene expression could be compared to the endogenous *actin* gene. The amplified PCR products were separated by 1.5% TAE-agarose gel electrophoresis in gels containing ethidium bromide for visualization.

### Southern blot analysis

Genomic DNA was extracted from 3 g of leaf tissue using the cetyltrimethylammonium bromide (CTAB) method [41], and 50 µg of genomic DNA was digested overnight, separated by 1% TBE-agarose gel electrophoresis and transferred to a positively-charged nylon membrane (BioDyne® A 0.45 µm; Pall Life Science VWR; Darmstadt, Germany) by capillary blotting in 10× SSC. The DNA was fixed by UV cross-linking. The membranes were prehybridized in SDS phosphate buffer (7% SDS, 50 mM phosphate buffer (pH 7.0), 2% blocking reagent (Roche, Mannheim, Germany), 50% formamide, 5× SSC, 0.1% sodium lauroyl sarcosinate) at 42°C for 2 h and probed with a DIG-labeled PCR fragment at 42°C overnight. Double-strand DIG-labeled DNA probes were prepared by PCR with construct-specific primers (Table 1) using the corresponding binary vectors as the template and the DIG DNA Labeling Kit (Roche Mannheim, Germany). The probes were denatured by boiling for 10 min before hybridization. Membranes were washed twice at room temperature with 2× SSC, 0.1% SDS for 15 min, and then twice with 0.1× SSC, 0.1% SDS at 68°C for 20 min. Signal detection with an alkaline phosphatase-conjugated anti-DIG antibody was carried out using the DIG Nucleic acid Detection Kit (Roche, Mannheim, Germany). Blots were exposed on Kodak Biomax light film (VWR, Darmstadt, Germany).

### Enzyme-linked immunosorbent assay (ELISA)

Leaf samples (150 mg) were homogenized in liquid nitrogen and resuspended in 250 µl cold protein extraction buffer (250 mM sucrose, 50 mM Tris (pH 7.5), 1 mM EDTA, 2 mM PMSF, 0.1% Triton X-100). For seed samples, 150 mg homogenized seeds were resuspended in 500 µl extraction buffer. Samples were centrifuged for 10 min in a cooled bench-top centrifuge and the protein concentration in the supernatant was measured according to the Bradford (1976) method using Pierce reagent with bovine serum albumin (BSA) as the standard (Thermo scientific, Bonn, Germany).

Recombinant C5a was quantified using a commercial Human Complement Component C5a DuoSet ELISA (catalog no. DY2037; R&D Systems, Wiesbaden-Nordenstadt, Germany) according to the manufacturer's instructions. Briefly, 96-well plates were coated with a mouse anti-human C5a-specific antibody at a final concentration of 1 µg/ml at room temperature overnight. Following five washes with PBS containing 0.05% Tween-20, the plates were incubated with 100 µl diluted leaf extract at room temperature for 2 h. After another wash, the plates were incubated with the corresponding biotinylated detection antibody at room temperature for 1 h, washed again and incubated with streptavidin conjugated to horseradish peroxidase at room temperature for 30 min. Finally, the plate was incubated with tetramethylbenzidine (TMB) at room temperature for 15 min in the dark. The reaction was stopped with 250 mM sulfuric acid. Extinction was measured at 450 nm in a Synergy HT multidetection reader (Bio-Tek, Bad Friedrichshall, Germany).

### Western blot analysis

Total soluble protein was extracted from leaf and seed samples as described above and 100 µg of protein was resuspended in 1× sample buffer containing 10% glycerol, 150 mM Tris (pH 6.8), 3% SDS, 1% β-mercaptoethanol and 2.5% bromophenol blue. The samples were heated to 95°C for 5 min and separated under denaturing conditions by 15% SDS-PAGE and then electrophoretically transferred to a 0.45-µm PVDF membrane (VWR; Darmstadt, Germany). The proteins were transferred at a constant 2 mA/cm^2^ at room temperature for 2 h in a Bio-Rad Trans-Blot semi-dry transfer cell using 50 mM Tris, 40 mM glycine, 0.01% SDS and 20% methanol as the transfer buffer (pH 8.5). The membrane was blocked using PBS containing 0.05% Tween-20 and 5% nonfat milk powder at room temperature overnight and was then probed at room temperature for 2 h with a mouse monoclonal anti-human-C5a antibody (catalog no. MA1-25341, ABR BioReagents, USA) or rabbit polyclonal anti-human C5a antibody (catalog no. 5995-100; BioVision, Milpitas, USA), each at 1∶1000 dilution at room temperature for 2 h. After three washes, the membrane was probed at room temperature for 2 h with a HRP-conjugated secondary antibody, either goat anti-mouse (catalog no. 715-035-151; Dianvova, Hamburg, Germany) or donkey anti-rabbit (catalog no. NA934V; GE Healthcare, Munich, Germany), each at 1∶10000 dilution. The signal was detected by ECL chemiluminescence and the membrane was exposed to Kodak Biomax light X-ray film (VWR; Darmstadt, Germany) for 1 min before it was developed and fixed. Carrier-free recombinant human C5a produced in *E. coli* (catalog no. 2037-C5-025/CF, B&D Systems, Heidelberg, Germany) was used as a standard.

### Determination of C5a biological activity

C5a activity was detected using a rat basophilic leukemia (RBL) cell-line transfected with human C5aR was used, which was developed by Ali *et al*. [42]. The cell line was provided by MBM ScienceBridge GmbH (Göttingen, Germany). The bioassay for lysosomal enzyme secretion was conducted as described by Goldstein and Weissmann [43]. The amount of antigenic C5a, applied to the bioassay, was calculated on the basis of the C5a-specific ELISA, which detects the number of antigenic C5a per ml. The concentration was defined as ng C5a equivalents/ml. After incubation with recombinant C5a, the cells are induced to secrete N-acetyl-β-D-glucosaminidase which releases 4-nitrophenolate from the substrate 4-nitrophenyl-N-β-D-glucosamide. The photometric detection of 4-nitrophenolate indicates the concentration of active recombinant protein, which can then be defined as an EC_50_ value. Carrier-free recombinant human C5a produced in *E. coli* (catalog no. 2037-C5-025/CF, B&D Systems, Heidelberg, Germany) was used as a standard.

### Statistical methods

Statistical comparisons were carried out using the *F*-test (ANOVA including the Bonferroni post-hoc test) with *p*≤0.05 (two-sided) considered significant. The variability of different events and siblings was characterized by the relative coefficient of variation (CV, %) 
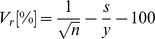
. The experimental design was calculated with SPSS.

## Results

### Stable transformation of *N. tabacum* cv. Geudertheimer

**Figure 1:**
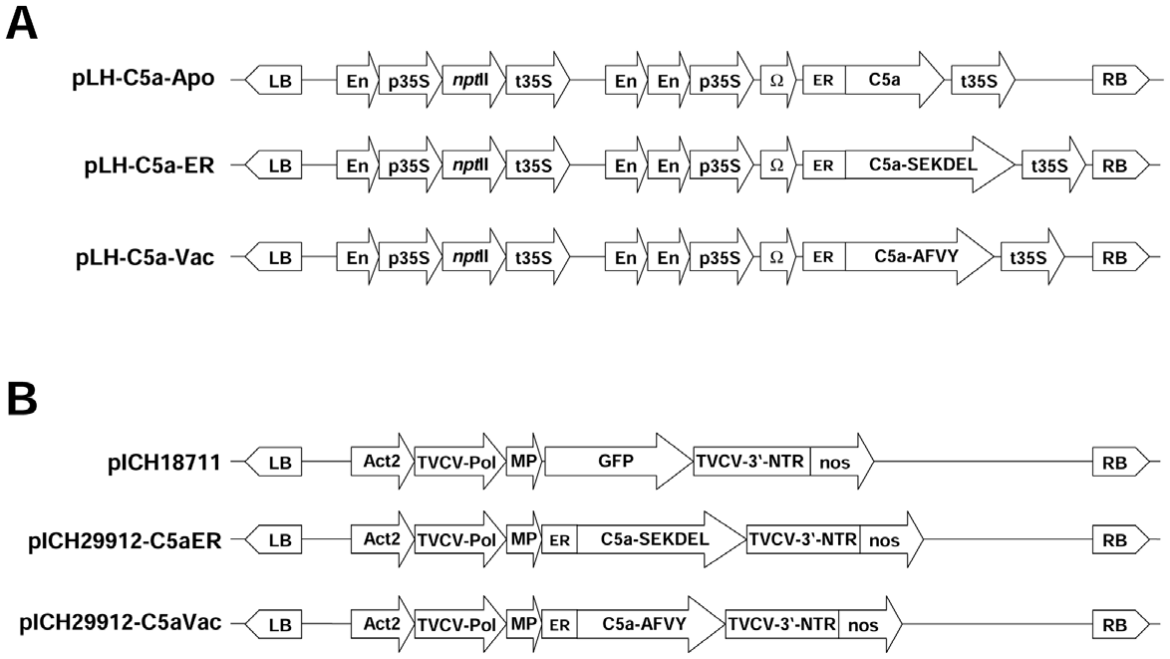
Schematic representations of the T-DNA constructs used to express human C5a in tobacco; (A) vectors used for stable transformation targeting the recombinant protein to different subcellular compartments. LB: left border; RB: right border; En: −340 bp to −91 bp CaMV 35S enhancer; p35S: −90 to −1 bp CaMV 35S core promoter; t35S: CaMV 35S terminator; Ω: 5′ *Tobacco mosaic virus* (TMV) untranslated region; *npt*II: neomycin phosphotransferase gene; ER: codon optimized signal peptide from the human IL6 gene; C5a: synthetic human C5a gene, codon optimized for tobacco; SEKDEL: ER retention motif; AFVY: vacuole-targeting peptide from common bean phaseolin protein; (B) Binary ‘MagnICON’ vectors used for transient expression. TVCV-Pol: RNA-dependent RNA polymerase from *Turnip vein clearing virus* (TVCV); MP: TMV movement protein; TVCV-3′-NTR: TVCV 3′ untranslated region; nos: *A. tumefaciens* nopaline synthetase gene terminator.

The C5a expression vectors for stable expression in tobacco (Fig. 1a) were constructed by incorporating the codon-optimized mature C5a sequence derived from the human C5 pro-protein gene and fusing this to the codon-optimized N-terminal ER-targeting peptide sequence from the human IL6 gene (Fig. S1, S2, S3). Three different variants were created to target the recombinant protein to the apoplast, ER and vacuole. The integrity of all the transformation vectors was verified by DNA sequencing (data not shown).

Putative T_0_ transgenic plants (grown under kanamycin selection) and non-transgenic controls were tested for the presence on the transgene by PCR on total genomic DNA from the crude leaf extracts, using specific primers (table 1). Transcription was verified by multiplex RT-PCR using the endogenous *actin* housekeeping gene as an internal control. Products of the expected size were detected in transgenic leaf tissue. The presence of the expected actin cDNA amplification product and the absence of the corresponding genomic product (1.1 kb) confirmed the absence of genomic DNA contamination (data not shown).

### Accumulation of C5a in the leaves and seeds of T_0_ transgenic plants

**Figure 2:**
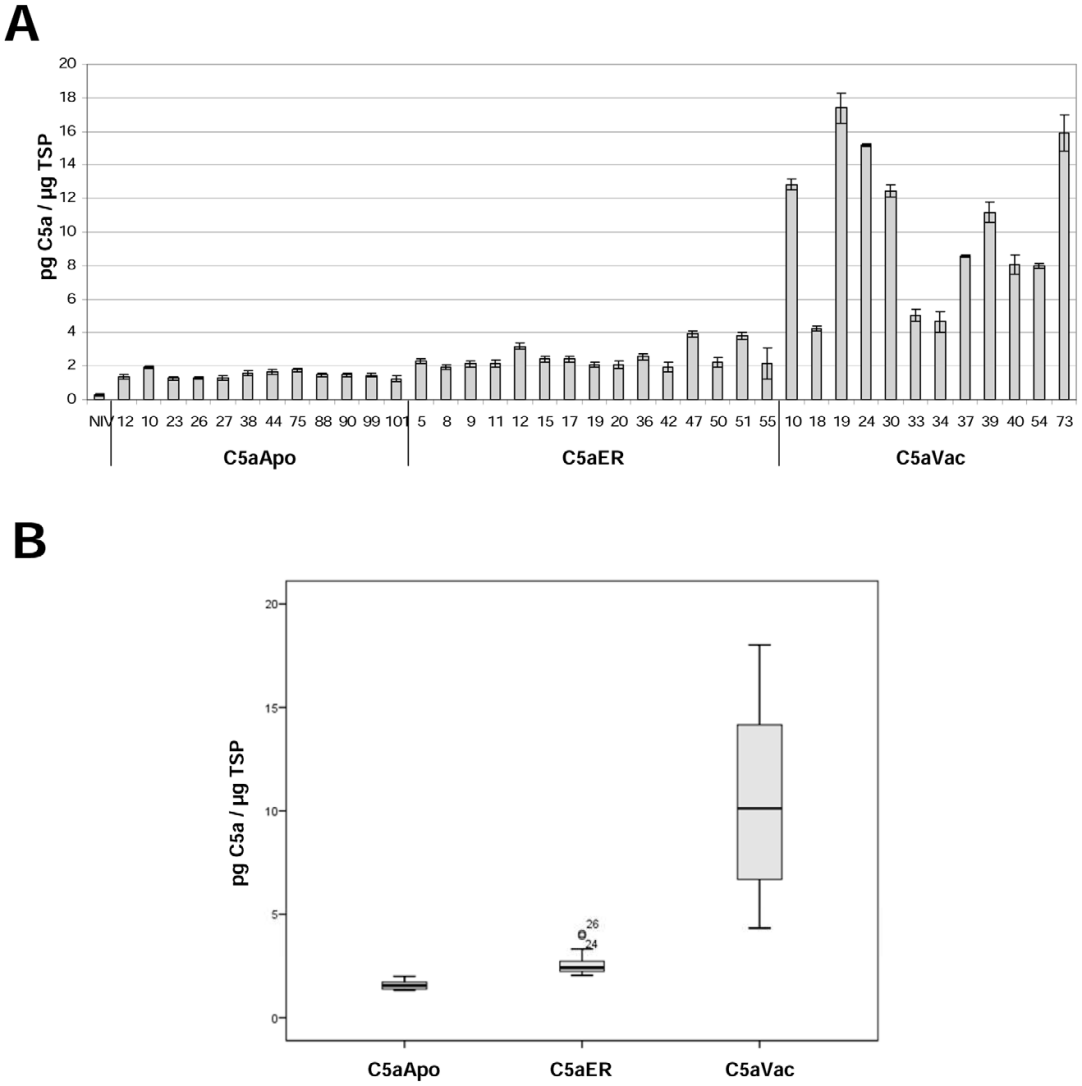
Protein expression levels in leaf extracts from T_0_ transgenic plants determined using at least two ELISAs. (A) Expression level measured in the individual transformants; Lane numbers indicate different independent tobacco events (NIV = wild-type control). (B) Box plot representation of C5a accumulation in the leaves of T_0_ transformants following ANOVA (including the Bonferroni post-hoc test).

Crude extracts from the uppermost fully-expanded leaves of 6-week-old tobacco plants in the greenhouse were used to determine C5a expression levels by ELISA. The highest yields were achieved by targeting to the vacuole (Fig. 2a). The average yield was 10.6 pg C5a per µg total soluble protein (TSP) and the best-performing event (C5aVac 19) produced 17.4 pg/µg TSP, equivalent to 1.49 µg C5a per gram fresh leaf weight (Table 2). The average yield of C5a was approximately an order of magnitude lower in the apoplast and ER variants, respectively 1.6 and 2.9 pg/µg TSP. We determined the coefficient of variation (CV) to measure the degree of variability between individual transgenic events (Fig. 2b). This was highest for the C5aVac construct (CV = 12.66%) whereas both C5aApo (CV = 3.86%) and C5aER (CV = 6.38%) showed relatively low variability.

**Table 2:** Expression levels in leaves and seeds of the best-performing line C5aVac 19 and transiently expressed C5aVac, using either TSP or fresh weight as the reference parameter.

Construct	Stably transformed line C5aVac 19						Transient expression of C5aVac
Generation	T_0_		T_1_		T_2_		
Individual	19		19-13		19-13-1		
Organ	leaf	seed	leaf	seed	leaf	seed	leaf
**C5a/TSP**	17.4	18.5	132.8	143.2	140.3	121.7	6985.8
**[pg/µg]**							
**C5a/fresh weight**	1.49	4.63	10.62	35.80	11.22	30.43	558.86
**[µg/g]**							

In order to investigate the accumulation of C5a in seeds and subsequent generations of plants, the ten best-performing T_0_ plants representing each construct were self-pollinated allowing the collection of T_1_ seeds. Pools of mature seeds weighing 150 mg were harvested from each T_0_ event, and extracts of total soluble protein were analyzed by ELISA.

**Figure 3:**
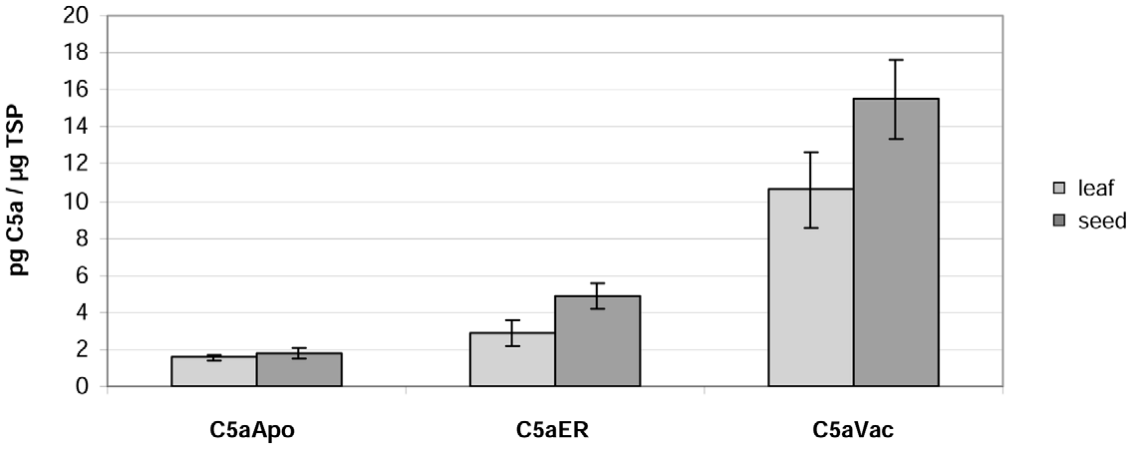
Average protein levels in the leaves and seeds of ten transgenic T_0_ tobacco plants expressing different C5a variants, using TSP as the reference parameter, based on at least two independent ELISAs.

**Figure 4:**
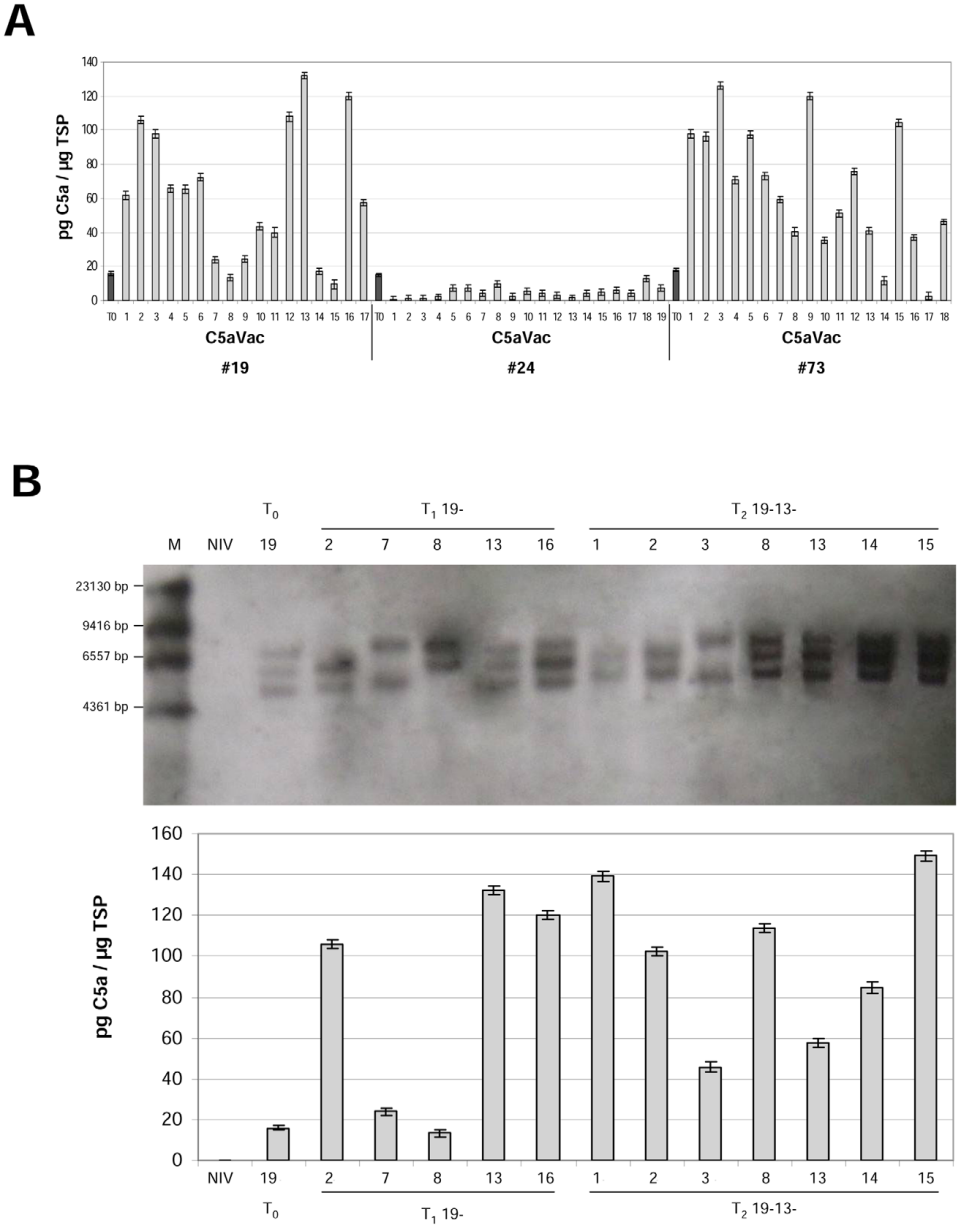
Protein expression levels in the T_1_ generation of three independent transgenic tobacco lines accumulating C5a in the vacuole, as determined by at least two independent ELISAs. (A) Expression level measured in the individual descendants; Lane numbers represent the different T_1_ individuals and T_0_ represents the parent. (B) Southern blots of the C5aVac 19 T_0_ parent and selected T_1_ and T_2_ progeny (identified by lane numbers) and corresponding C5a expression levels determined by ELISA. The genomic DNA was digested with HindIII, which cuts once in the expression vector, and the presence of segregating bands indicates of three unlinked loci in the T_0_ parent. M = DIG-labeled DNA Molecular Weight Marker II (Roche), NIV DNA from wild-type negative control plant.

Using TSP as the reference parameter (Fig. 3), the average yield of C5aApo was 1.8 pg/µg TSP, which is similar to the level detected in T_0_ leaves. The yield of C5aER was approximately 1.7-fold higher in seeds (4.9 pg/µg TSP compared to 2.9 pg/µg TSP in leaves; Fig. 4a) and that of C5aVac was approximately 1.5-fold higher in seeds (15.5 pg/µg TSP compared to 10.6 pg/µg TSP in leaves; Fig. 3). The difference between leaves and seeds was less striking in the higher-performing plants, e.g. the yield of C5a in the T_1_ seeds of the best-performing line C5aVac 19 (18.5 pg/µg TSP) was only slightly higher than the yield in leaves (17.4 pg/µg TSP) as shown in table 2.

Using the fresh tissue weight as the reference parameter, C5a yields were approximately four times higher in seeds than leaves, regardless of the targeting strategy. Using C5aVac 19 as example, the average yield in leaves was 1.49 µg/g fresh weight, compared to 4.63 µg/g in seeds (Table 2). The CV values for the variability of expression among different lines were similar to those observed in leaves.

Dry T_1_ seeds were stored at room temperature for three months and the C5a levels were determined again by ELISA, revealing no significant change in abundance compared to freshly-harvested seeds (data not shown).

### Accumulation of C5a in the leaves and seeds of subsequent generations of plants

Descendants of the three best-performing T_0_ transformants (C5aVac 19, 24, 73; see Fig. 3) were self-pollinated to obtain transgenic plants up to the T_2_ generation, which were selected on kanamycin to ensure the locus remained active. Most of the T_1_ descendants of C5aVac 19 and C5aVac 73 accumulated more C5a than their parents, but there was no generational increase in line C5aVac 24 (Fig. 4a). The expression levels among T_1_ siblings were relatively heterogeneous, with CVs of 15.08%, 13.64% and 12.66% for lines C5aVac 19, 24 and 73, respectively. Some T_1_ plants accumulated less recombinant protein than their parent (e.g. C5aVac 19-15, which produced 10.6 pg/µg TSP) whereas others produced substantially more (e.g. the best-performing plant C5aVac 19-13, which produced 132.8 pg/µg TSP, corresponding to 10.62 µg/g fresh leaf weight (Table 2)).

C5aVac 19-13 was self-pollinated and C5a levels were measured in the T_2_ seeds. The average expression level was 143.2 pg/µg TSP or 35.8 µg/g fresh weight (Table 2) which represents a near eight-fold increase in seeds over one generation. Only two individual T_2_ plants (C5aVac 19-13-1 and 19-13-15) performed better that their T_1_ parents (Fig. 4b). Leaves from the best-performing plant (C5aVac 19-13-1) produced 140.3 pg/µg TSP or 11.22 µg/g fresh leaf weight (Table 2). The expressions level among the T_2_ siblings were less heterogeneous (CV of 7.17) compared to the T_1_ individuals (CV of 15.08). The yield of C5a in the leaves of line C5aVac 19 increased by an order of magnitude between the T_0_ and T_1_ generations but there was only a marginal increase in the T_2_ generation (18.3→132.8→140.3 pg/µg TSP) was not significant. The accumulation of C5a in the seeds of plant C5aVac 19-13-1 (121.7 pg/µg TSP or 30.4 µg/g fresh seed weight) was slightly lower compared its T_1_ parent (143.2 pg/µg TSP). Most T_2_ descendants of C5aVac 19-13 accumulated less C5a than the T_1_ parent, although the variability in expression levels was lower (CV = 7.17% for T_2_ compared to 15.08% for T_1_).

### Transgene insertions in the lines C5aVac 19, 24 and 73

We analyzed the transgene locus structure in T_0_ plants from lines C5aVac 19, 24 and 73, and in T_1_ progeny with the highest and lowest expression levels between the corresponding siblings (Fig. 4a), namely 19-2, -7, -8, -13, -16 and 24-4, -6, -8, -13, -16 and 73-3, -9, -10, -14, -15. We further analysed the T_2_ individuals 19-13-1, -2, -3, -8, -13, -14, -18 (Fig. 4b). We detected three transgene loci in the T_0_ plant C5aVac 19, and the two best-performing T_1_ plants from this line (C5aVac 19-13 and 19-16) retained all three loci whereas the other three lines (C5aVac 19-2, 19-7 and 19-8) retained only two loci (Fig. 4b). In the T_2_-generation the highest expression was observed in plants with all three but also with only two loci. As expected for lines with high copy number, the segregation analysis of C5aVac 19; 19-2; 19-7; 19-8; 19-13; 19-16; 19-13-1; 19-13-15 revealed always nearly 100% transgenic offspring. Hence no correlation between the number of transgene loci and the performance of individual plants was detectable. There were also three transgene loci in the T_0_ plant C5aVac 73 and these were retained in all five T_1_ plants we tested (data not shown). Given the highly variable expression levels among these T_1_ plants, the locus structure did not appear to affect the performance of individual plants. There was a single locus in T_0_ plant C5aVac 24 and in all five T_1_ individuals we tested (data not shown).

### Transient expression of C5aER and C5aVac in *N. benthamiana*, using the MagnICON system

**Figure 5:**
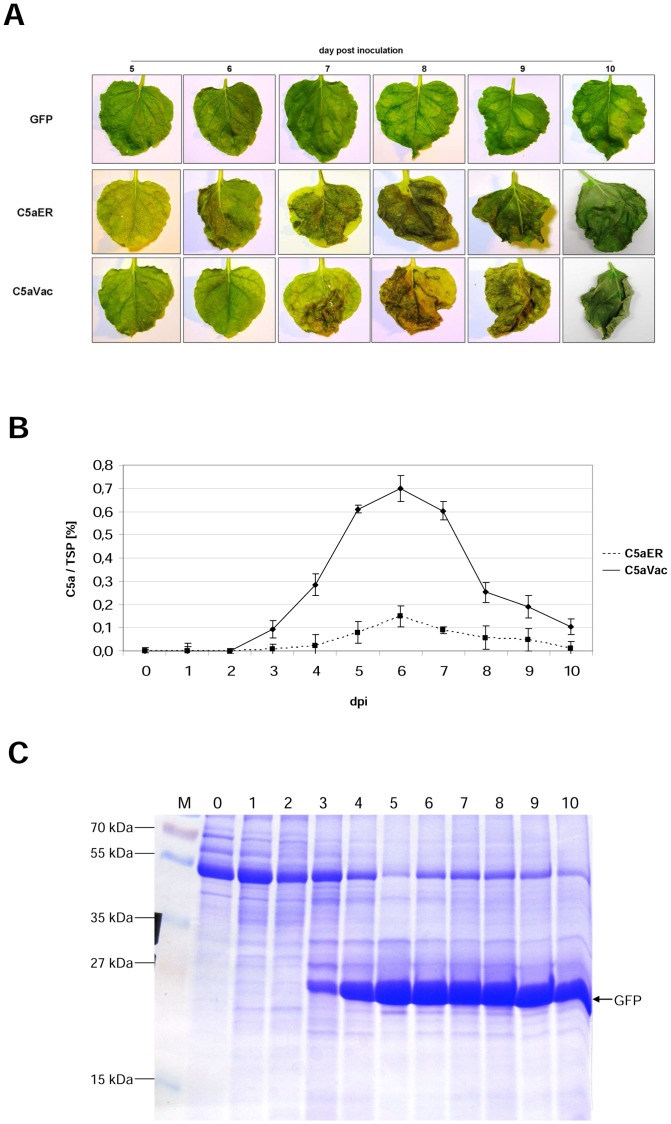
Leaves of *N. benthamiana* Agroinfiltrated with either GFP-, C5aER- or C5aVac-expressing vectors pICH18711, pICH29912-C5aER and pICH29912-C5aVac, respectively. One representative of at least three independent experiments is shown. (A) Pictures of leaves made under UV-light prior to sampling. dpi: days post inoculation (B) Yield of recombinant C5a as percentage of TSP in Agroinfiltrated leaves of *N. benthamiana*, sampled from 0–10 dpi. (C) Coomassie-stained SDS-PAGE of leaf extracts N. benthamiana leaves, Agroinfiltrated with GFP-expressing control vector pICH18711, sampled from 0–10 dpi.

For transient expression, the coding regions of C5aER and C5aVac were introduced into the MagnICON vector pICH29912 (Fig. 1b), which was kindly provided by Prof. Dr. Yuri Gleba and Dr. Anatoli Giritch (Nomad Bioscience; Halle/Saale, Germany). We selected these two C5a variants, since in stable transformed plants, the C5aApo and C5aER led to similarly low levels of recombinant protein while C5aVac revealed significantly higher yield. The tobacco cultivar Geudertheimer, which was used for stable transformation, has been recorded to be not amenable for the MagnICON system [44]. Therefore, we expressed both genes transiently in *N. benthamiana*, which is commonly used for transient expression assays and known to promote high level expression of viral replicons [45], [46]. However, overexpression of both C5a variants was accompanied by cytotoxic effects and induced leave senescence whereas no severe phenotype was observed in plants infected with the GFP control vector pICH18711 (Fig. 5a). Moreover, we observed a rapid decline in the yield of the target protein in the dying leaves (Fig. 5b). Nevertheless, the highest accumulation level measured at 6 dpi at the beginning leaf senescence reached up to 0.7% of TSP for C5aVac and 0.2% of TSP for C5aER (Table 2). The difference in the accumulation level between transiently expressed C5aER and C5aVac was similar to the one observed in stable transformed tobacco.

### Molecular and functional characterization of recombinant plant-derived C5a

**Figure 6:**
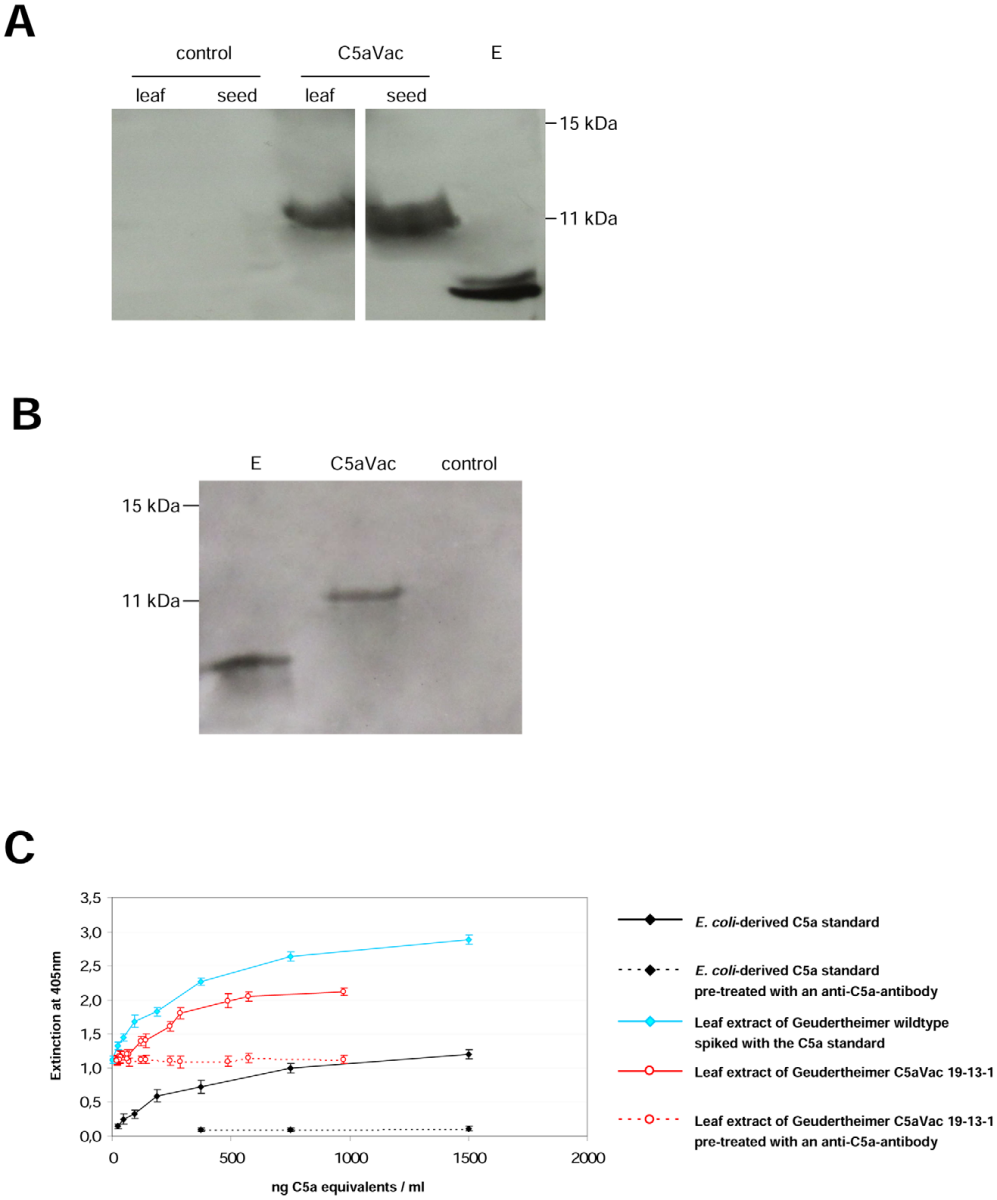
Western Blot analysis of leaf and seeds samples of transgenic Geudertheimer plants (100 µg TSP); (A) Samples from stable transformed plants; E: 5 ng of recombinant C5a produced in *E. coli*; Control: leaf and seed samples from wild-type tobacco. C5aVac: leaf and seed samples of the T_2_ individual C5aVac 19-13-15, expressing the vacuolar variant of C5a; (B) Samples from *N. benthamiana* at 6 dpi after Agroinfiltration; control: *N. benthamiana* transfected with the empty vector pICH29912; C5aVac: *N. benthamiana* transfected with pICH29912-C5aVac; E: 5 ng of recombinant C5a produced in *E. coli*; (C) Verification of C5a biological activity using crude leaf extracts from transgenic tobacco and the near isogenic variant (wildtype) compared to a commercial standard C5a produced in *E. coli.* Enzyme release was measured by substrate conversion following exposure to different dilutions of the lead extracts, with the concentration of C5a determined by ELISA.

Western blot analysis of TSP extracts from leaf and seeds samples of transgenic C5aVac plants (Fig. 6a) and transfected *N. benthamiana* plants (Fig. 6b) showed a distinct band that migrates at 12 kDa, which is larger than the recombinant product from *E. coli* (9 kDa) suggesting that the plant-derived recombinant protein might be glycosylated. Crude leaf extracts from the T_2_ individual C5aVac 19-13-1 and the near isogenic variant as control were used for C5a bioassays based on a RBL-cell line, transfected with an C5a-receptor. The cell line releases an enzyme upon binding of biological active C5a which can be measured by substrate conversion at 405 nm. The concentration of C5a which induced the half-maximal enzyme release was determined as EC_50_ value. Interestingly, extracts of the near isogenic variant (niv) that served as control converted the substrate as proven by the incubation of mere leaf samples with the substrate (data not shown). Nevertheless, spiked with the C5a standard the NIV induced an additional enzyme release in a dose-dependent manner similar to the commercial C5a standard (Fig. 6c). Hence the basic level of conversion observed in the NIV without additional enzyme was subtracted from the values measured for the transgenic variants. The EC_50_ value of both samples was calculated at approximately 200 ng/ml. The different concentrations of C5a in the assays were adjusted based on the values measured by C5a-ELISA, in the transgenic leaf and seed extracts. Leaf extracts of C5aVac 19-13-1 induced enzyme release in a manner comparable to the C5a standard but the EC_50_ value of 279.48 ng/ml was higher (Fig. 6c). Samples of transgenic plants and seeds and from leaves derived from the transient expression in *N. benthamiana*, resulted in an equal EC_50_-value (data not shown). Incubating the transgenic leaf samples with the anti-C5a-antibody, used for the Western Blots, prior to the bioassay, reduced the response to the level of the NIV extract (Fig. 6c).

## Discussion

We have demonstrated for the first time that the human complement factor C5a can be produced in a soluble and active form in stable transformed tobacco, although the yields were low compared to other recombinant proteins expressed using the CaMV 35S promoter [19], [47]. This may reflect the fact that C5a is an unstable protein that is rapidly degraded in the plasma following its release from the precursor C5 [48], [49]. The degradation of recombinant proteins in plants is a relatively common occurrence that limits accumulation *in planta*, and it is possible that unstable human proteins are also susceptible to endogenous plant proteases [50], [51].

Targeting recombinant proteins to different tissues and subcellular storage compartments can significantly influence the protein stability and yield [27], [52]. In the case of C5a, the vacuole appears to be the most suitable storage compartment, since the highest yields were achieved in leaves and seeds when appending the recombinant protein with the PSV-specific targeting peptide AFVY (Fig. 2a, b). This result was unexpected in leaves because PSVs are underrepresented in vegetative tissues, whereas lytic vacuoles are prevalent that do not support protein accumulation [53]. However, it has been demonstrated that the expression of seed storage proteins in leaves can induce the formation of storage organelles in vegetative tissue [54].

Many heterologous proteins accumulate to higher levels when targeted for retention in the ER because this compartment contains abundant molecular chaperones to fold proteins correctly and maintain their solubility but there are few proteases [50]. This contrasts with the apoplast, where abundant proteases often cause significant degradation [50], [55]. Interestingly, we did not find a significant difference between these compartments and C5a accumulation was an order of magnitude lower than in the vacuole. This suggests that C5a is specifically degraded in the ER because it is sensitive to the limited number of highly-specific proteases contained therein. Recently, this phenomenon has also been recorded for other proteins recombinant proteins [56]–[58]. The higher yields of vacuolar C5a indicate that the recombinant protein may be partially protected by virtue of its vacuolar destination, perhaps due to a shorter residence time in the ER. The vacuole therefore appears to be an appropriate target for recombinant proteins expressed in plants if they are susceptible to proteases in the secretory pathway.

Seeds accumulated 1.4–1.7 more C5a than leaves when the protein was targeted to the ER or the vacuole, probably reflecting the lower proteolytic activity [50] and the higher protein concentration in seeds 25% compared to 10% in tobacco leaves [59], [60]. There was no difference between seeds and leaves when the protein was targeted to the apoplast, probably because this does not function as a storage compartment in either tissue. Interestingly, the only previous publication known to us that compares the accumulation of proteins in tobacco leaves and seeds using the CaMV 35S promoter, reported yields of up to 5% of TSP in leaves and only 0.4% in seeds [61]. Since the transgene encoded protein is a relatively stable antibody, this difference might reflect the promoter activity, which is known to be less active in seeds compared to leaves [62]. Assuming, that C5a is more stable in seeds compared to leaves this might balance the lower activity of the promoter.

We noted a sharp increase in protein accumulation in both leaves and seeds between the T_0_ and T_1_ generations, which might reflect homozygosity of the transgene loci for the individuals 19-13 and 19-16 since the same integration loci are present. Phenomena like these have already been demonstrated [63]. However, the increase seems to be too pronounced to reflect only the doubling of the copy number. In addition, since all offspring of a selfed homozygous line should be homozygous, the T_2_ generation should show similar expression levels, which is not the case. Hence other factors might influence the increase in transgene expression, which has also been reported by others [64], [65].

The protein levels in the seeds were stable for more than three months providing a strong advantage over leaves, where proteins degrade soon after harvest and must be extracted promptly [50]. Tobacco seeds also lack many of the secondary metabolites that interfere with and increase the costs of downstream processing [66]. Morandini *et al*. [67] calculated that Arabidopsis is a realistic seed platform for the production of vaccines which are needed in moderate amounts, considering several growth cycles per year in the greenhouse, the high TSP content per fresh weight and the high expression levels of the recombinant protein above 1% TSP. Therefore tobacco, which provides comparable seeds yields, might also be suitable for the commercial production of recombinant C5a. The fact that the maximum yield we achieved in plants (0.014% of TSP) falls some way short of the 0.65% of TSP achieved in *E. coli* [17] might possibly be improved by using strong seed-specific promoters and the use of other cultivars like maize, rice, barley and safflower which are currently the seed-based systems of choice for the expression of pharmaceuticals [68]. It remains to be seen if yields can be improved to the extent that the process becomes commercially competitive with microbial fermentation.

As alternative to stable transformed plants, transient expression systems are likely to be used for the commercial production of biopharmaceuticals because they can be established and scaled more rapidly than transgenic plants [23]. Using MagnICON vectors, C5aER and C5aVac reached up to 0.2% and 0.7% of TSP, respectively, in *N. benthamiana*. In case of C5aVac this represents a 50fold increase compared to the best-performing transgenic T_2_ individual C5aVac 19-13-15. The prodigious synthesis rate of the viral vector seems to be able to overcome the low accumulation levels that probably results from protein degradation. This phenomenon has already been recorded for other unstable proteins, including the hepatitis B virus surface antigen (HbsAg), which accumulated at less than 0.01% TSP in transgenic tobacco leaves but up to 0.26% TSP using the MagnICON system [69], and the LTB-MUC1 protein which achieved yields of 3% TSP by transient expression [70]. Notably, the same difference between C5aER and C5aVac as observed in stable transformed Geudertheimer plants was recorded in the transient assay as well. However, transient overexpression in *N. benthamiana* was accompanied by cytotoxic effects, which induced a premature leaf senescence for both constructs, starting at 5 dpi. Moreover, we recorded a rapid decrease of recombinant C5a in the dying leaves. Similarly, the transient expression of LTB-MUC1 and the hepatitis B core antigen led to complete leaf necrosis [70], [71]. Pinkhasov *et al*. [70] speculated that the recombinant protein might interfere with the plant metabolism and cause altered phenotype or cell death. This is more evident in the MagnICON viral vector system because of extremely high accumulation levels. Nevertheless, the reason for C5a-induced leaf necrosis remains unknown.

The C5a isolated from tobacco had a significantly higher molecular weight than the *E. coli* standard (Fig. 6). This might reflect the addition of *N*-linked glycans at the Asn_64_ residue, which is also glycosylated in the native human protein [72]. In fact, the potential *N*-glycosylation sites were found to be conserved between plants and animals [73]–[75]. The assumption that plant derived C5a is glycosylated is also supported by the fact that, the EC_50_ value of plant-derived C5aVac of 279.46 ng C5a equivalents/ml was different compared to that of *E. coli*-derived counterpart with 192.03 ng C5a equivalents/ml [17]. Using the plant extract spiked with *E. coli* derived C5a we could prove that this difference is not due to endogenous metabolites or enzymes present in the crude extract. Nevertheless, additional experiments need to be done in order to prove that plant-derived C5a is glycosylated.

## Supporting Information

Figure S1 **Sequence of the IL6 signal peptide including three triplets downstream the initiator codon ATG (underlined) that increase the efficiency of recognition.**
(DOC)

Figure S2 **Sequence of the mature C5a gene product, derived from the precursor C5.**
(DOC)

Figure S3 **Compartment specific C-terminal variants of C5a codon-optimized for tobacco.**
(DOC)
