# Correction: Expression and Subcellular Targeting of Human Complement Factor C5a in *Nicotiana species*

**DOI:** 10.1371/annotation/0e288695-705d-4363-aa02-2239a55ddeff

**Published:** 2013-05-17

**Authors:** Henrik Nausch, Heike Mikschofsky, Roswitha Koslowski, Udo Meyer, Inge Broer, Jana Huckauf

The second author's name is misspelled. The correct spelling is: Heike Mikschofsky

The correct citation is:

Nausch H, Mikschofsky H, Koslowski R, Meyer U, Broer I, et al. (2012) Expression and Subcellular Targeting of Human Complement Factor C5a in Nicotiana species. PLoS ONE 7(12): e53023. doi:10.1371/journal.pone.0053023 

