# Supplementary material for: Expression and Subcellular Targeting of Human Complement Factor C5a in Nicotiana species
Source: PLoS One. 2012 Dec 28;7(12):e53023. doi: 10.1371/journal.pone.0053023 (PMC3532468; doi:10.1371/journal.pone.0053023)
Supplement: Figure S1 — Sequence of the IL6 signal peptide including three triplets downstream the initiator codon ATG (underlined) that increase the efficiency of recognition. (DOC) [file pone.0053023.s001.doc]

**Fig. S1:**

M A S S N S F S T S A F G P 14

ATG GCT TCC TCC AAT TCA TTC TCT ACT TCG GCC TTT GGT CCA 42

V A F S L G L L L V L P A A 28

GTT GCA TTC AGC CTT GGA TTG CTC TTA GTG TTG CCT GCT GCA 84

F P A 31

TTT CCT GCG 93
