# Supplementary material for: Expression and Subcellular Targeting of Human Complement Factor C5a in Nicotiana species
Source: PLoS One. 2012 Dec 28;7(12):e53023. doi: 10.1371/journal.pone.0053023 (PMC3532468; doi:10.1371/journal.pone.0053023)
Supplement: Figure S2 — Sequence of the mature C5a gene product, derived from the precursor C5. (DOC) [file pone.0053023.s002.doc]

**Fig. S2:**

T L Q K K I E E I A A K Y K 46

ACT CTC CAG AAG AAG ATC GAA GAG ATT GCA GCT AAG TAT AAA 135

H S V V K K C C Y D G A C V 61

CAC AGT GTT GTG AAG AAG TGT TGC TAC GAC GGA GCT TGT GTT 177

N N D E T C E Q R A A R I S 75

AAT AAC GAT GAG ACC TGC GAA CAG AGA GCA GCT AGG ATA TCT 219

L G P R C I K A F T E C C V 89

CTT GGT CCA AGA TGT ATT AAA GCC TTC ACA GAG TGC TGT GTC 261

V A S Q L R A N I S H K D M 103

GTT GCT TCA CAA TTG CGT GCT AAT ATC TCT CAT AAG GAT ATG 303

Q L G R L 108

CAA CTT GGC AGA CTG 318
