# Supplementary material for: Expression and Subcellular Targeting of Human Complement Factor C5a in Nicotiana species
Source: PLoS One. 2012 Dec 28;7(12):e53023. doi: 10.1371/journal.pone.0053023 (PMC3532468; doi:10.1371/journal.pone.0053023)
Supplement: Figure S3 — Compartment specific C-terminal variants of C5a codon-optimized for tobacco. (DOC) [file pone.0053023.s003.doc]

**Fig. S3:**

**Apoplasma:** * 218/108

TAA 648/318

**ER-Retention Signal:** S E K D E L * 224/114

TCT GAG AAG GAT GAG CTT TAA 666/336

**Vacuole Sorting Signal:** A F V Y * 222/112

GCT TTC GTG TAC TAA 660/330
